# Targeting transmembrane-domain-less MOG expression to platelets prevents disease development in experimental autoimmune encephalomyelitis

**DOI:** 10.3389/fimmu.2022.1029356

**Published:** 2022-10-27

**Authors:** Yuanhua Cai, Jocelyn A. Schroeder, Weiqing Jing, Cody Gurski, Calvin B. Williams, Shaoyuan Wang, Bonnie N. Dittel, Qizhen Shi

**Affiliations:** ^1^ Department of Pediatrics, Medical College of Wisconsin, Milwaukee, WI, United States; ^2^ Blood Research Institute, Versiti, Milwaukee, WI, United States; ^3^ Fujian Institute of Hematology, Fujian Provincial Key Laboratory on Hematology, Fujian Medical University Union Hospital, Fuzhou, China; ^4^ Department of Microbiology and Immunology, Medical College of Wisconsin, Milwaukee, WI, United States; ^5^ Children’s Research Institute, Children’s Wisconsin, Milwaukee, WI, United States; ^6^ Midwest Athletes Against Childhood Cancer (MACC) Fund Research Center, Milwaukee, WI, United States

**Keywords:** immune tolerance induction, experimental autoimmune encephalomyelitis, gene therapy, platelet-targeted, MOG (myelin oligodendrocyte glycoprotein)

## Abstract

Multiple sclerosis (MS) is a chronic inflammatory autoimmune disease of the central nervous system with no cure yet. Here, we report genetic engineering of hematopoietic stem cells (HSCs) to express myelin oligodendrocyte glycoprotein (MOG), specifically in platelets, as a means of intervention to induce immune tolerance in experimental autoimmune encephalomyelitis (EAE), the mouse model of MS. The platelet-specific αIIb promoter was used to drive either a full-length or truncated MOG expression cassette. Platelet-MOG expression was introduced by lentivirus transduction of HSCs followed by transplantation. MOG protein was detected on the cell surface of platelets only in full-length MOG-transduced recipients, but MOG was detected in transmembrane-domain-less MOG_1-157_-transduced platelets intracellularly. We found that targeting MOG expression to platelets could prevent EAE development and attenuate disease severity, including the loss of bladder control in transduced recipients. Elimination of the transmembrane domains of MOG significantly enhanced the clinical efficacy in preventing the onset and development of the disease and induced CD4^+^Foxp3^+^ Treg cells in the EAE model. Together, our data demonstrated that targeting transmembrane domain-deleted MOG expression to platelets is an effective strategy to induce immune tolerance in EAE, which could be a promising approach for the treatment of patients with MS autoimmune disease.

## Introduction

Inducing antigen-specific immune tolerance is desirable in autoimmune diseases. Multiple sclerosis (MS) is a chronic inflammatory autoimmune disease associated with gradual degeneration of myelination in the central nervous system (CNS), resulting in neurological decline with paresis and eventually disability. Currently, there is no cure for MS. Suppression of inflammatory activity is the basic treatment option. This treatment reduces disease progression and clinical relapse but is not curative and requires long-term disease-modifying therapy, which is often associated with severe complications (1–4). While immune reconstitution (IR) therapies utilizing chemotherapies, monoclonal antibodies, or autologous hematopoietic stem cell (HSC) transplantation (HSCT) may induce disease remission and prevent relapses, IR therapies are still limited by morbidity and mortality (5–8). Thus, it is desired to develop new therapeutic approaches that can prevent or reverse MS.

While the exact pathogenic mechanism of MS development is still unclear, evidence suggests that inflammation initiated by antigen-specific T cells leads to disability in patients with MS (9). Myelin oligodendrocyte glycoprotein (MOG) is expressed by oligodendrocytes and plays a role in the myelination of nerves in the CNS. Demyelination occurs during MS development and contributes to the multitude of symptoms associated with MS pathogenesis (10–13). The mouse model of MS, experimental autoimmune encephalomyelitis (EAE), has been widely used for MS studies as they share similar clinical and pathophysiological features (9). EAE, which is characterized by inflammation leading to demyelination and neuronal damage, can be induced by immunization with the MOG_35-55_ peptide (9). Antigen-specific immune tolerance induction *via* gene therapy is an attractive emerging approach for the treatment of patients with MS (14, 15).

Platelets are a unique target for gene therapy to induce immune tolerance due to their innate protein storage and release capacity, expression of immunomodulatory molecules and cytokines, natural turnover with a life-span of approximately 5 days in mice and 10 days in humans, and their ability to interact with various cells of the immune system (16–22). Our previous studies have demonstrated that targeting coagulation factor VIII (FVIII) expression to platelets under the control of the platelet-specific αIIb promoter (2bF8) results in the storage of FVIII in platelet α-granules and that platelet-derived FVIII can effectively induce antigen-specific immune tolerance in hemophilia A mice even with pre-existing anti-FVIII immunity (23–28). When a similar approach was applied to a hemophilia B model, which results from factor FIX (FIX) deficiency, antigen-specific immune tolerance was induced in hemophilia B mice even with anti-FIX immunity (29, 30). Furthermore, targeting non-coagulant protein ovalbumin (OVA) to platelets under the same promoter used for the FVIII and FIX studies also resulted in neoprotein OVA storage in platelet α-granules, leading to antigen-specific immune tolerance (31, 32).

In this study, we evaluated the potential of platelet-specific MOG expression in immune tolerance induction in an EAE model of autoimmune disease. We examined the impact of the cellular location of the neoprotein when MOG expression was targeted to platelets on the efficacy of immune tolerance induction. We showed that lentivirus-mediated platelet-specific MOG gene delivery to HSCs induced immune tolerance in EAE, and a transmembrane-domain-less truncated MOG significantly enhanced the efficacy in immune tolerance induction.

## Results

### MOG lentiviral vector construction and lentivirus verification in Dami cells

Our previous studies indicated that the subcellular location of the neoprotein expressed in platelets might impact immune tolerance induction in platelet-targeted gene therapy (33). MOG is a transmembrane protein expressed on the cell surface of oligodendrocytes that make up the myelin sheath in the CNS (34, 35). In this study, we made three MOG constructs, two of which contain truncated MOG without the transmembrane domain. Specifically, we investigated whether platelet-targeted MOG expression induced immune tolerance and compared the efficacy of immune tolerance induction between MOG proteins with different subcellular locations.

**Figure 1:**
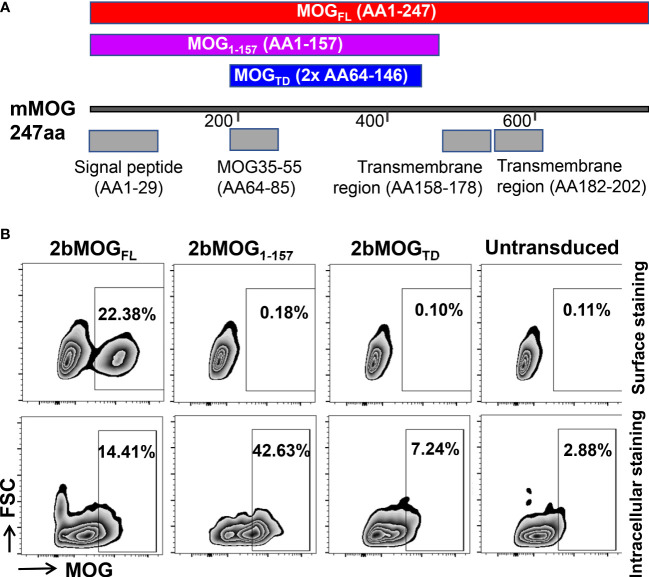
Generation and evaluation of platelet-specific MOG expression lentiviral vectors. **(A)** Schematic diagram of MOG_TD_, MOG_1-157_, and MOG_FL_ expression cassettes. Each MOG expression cassette was placed under the control of the platelet-specific αIIb promoter. **(B)** MOG expression in a promegakaryocyte cell line, Dami cells. Lentiviral vectors harboring the 2bMOG_TD_, 2bMOG_1-157_, or 2bMOG_FL_ expression cassette were produced by transient transfection of HEK293 cells. Dami cells were transduced with lentiviruses. After 72 hours of culture, cells were stained with anti-MOG antibody with or without permeabilization and analyzed by flow cytometry. Representative figures from flow cytometry analysis are shown.

Three expression cassettes (**Figure 1A**) were made to introduce MOG protein expression in our study. One was MOG tandem (MOG_TD_), which contains two copies of the MOG peptide from amino acids 64-146, including the 35-55 encephalitogenic sequence but excluding the two transmembrane regions and the signal peptide. The second one was MOG_1-157_, which contains a truncated MOG protein without the two transmembrane regions, but includes the signal peptide from amino acids 1 to 157. The third one was MOG_FL_, which contains full-length MOG protein from amino acids 1 to 247, including the transmembrane regions and signal peptide.

To validate the viability of each lentiviral vector that harbors a MOG expression cassette under the platelet-specific αIIb promoter, Dami cells, a human promegakaryocyte cell line (36), were transduced with 2bMOG_TD_, 2bMOG_1-157_, or 2bMOG_FL_ lentivirus. MOG protein expression was detected by flow cytometry. As shown in **Figure 1B**, using cell surface staining (no permeabilization), MOG neoprotein was detected on Dami cells transduced with the 2bMOG_FL_ lentivirus, but not on the cells transduced with either the 2bMOG_1-157_ or 2bMOG_TD_ lentivirus. Of note, when cells were permeabilized followed by MOG intracellular staining, MOG neoprotein was detected in all three groups (**Figure 1B**). These data demonstrate that 2bMOG_1-157_ and 2bMOG_TD_ can drive MOG intracellular protein expression, but only 2bMOG_FL_ can drive protein expression on the cell surface.

### Introducing MOG expression in platelets in mice

**Figure 2:**
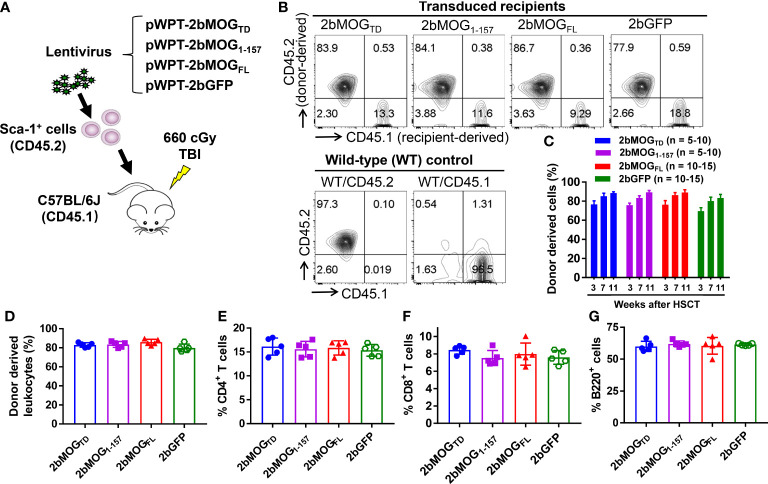
Targeting MOG expression to platelets did not affect the leukocyte profile. Sca-1^+^ HSCs/progenitors isolated from CD45.2 WT C57BL/6J donors were transduced with lentiviruses and transplanted into CD45.1 recipients preconditioned with 660 cGy total body irradiation. After HSCT and BM reconstitution, blood samples were collected from recipients at various time points, and leukocytes were stained for CD45.1, CD45.2, CD4, CD8, and B220. After staining, cells were analyzed by flow cytometry. Representative results from week 7 from one trial after HSCT are shown. **(A)** Schematic diagram of experimental design to generate 2bMOG_TD_, 2bMOG_1-157_, 2bMOG_FL_, and 2bGFP recipients. **(B)** Representative dot plots from flow cytometry analysis of chimerism. **(C)** Chimerism in transduced recipients at various time points. **(D)** The chimerism in transduced recipients. **(E)** The percentage of CD4 T cells in transduced recipients. **(F)** The percentage of CD8 T cells in transduced recipients. **(G)** The percentage of B220 cells in transduced recipients. There were no significant differences in chimerism, CD4, CD8, or B cells between 2bMOG-transduced groups and the 2bGFP control group. **(D-G)** Each data point represents one mouse. Three replicate experiments were performed.

To introduce MOG expression in platelets, 2bMOG_TD_, 2bMOG_1-157_, and 2bMOG_FL_ lentiviruses were used to transduce Sca-1^+^ bone marrow (BM) cells isolated from WT CD45.2 mice and transplanted into WT CD45.1 recipients that received an optimized non-myeloablative preconditioning regimen for immune tolerance induction in platelet gene therapy (25, 27, 31, 32), 660 cGy total body irradiation (TBI) (**Figure 2A**). 2bGFP lentivirus (31) was used as an unrelated control vector in parallel. After BM reconstitution, blood samples were collected for flow cytometry to analyze the engraftment of donor-derived cells (**Figure 2B**). Donor-derived leukocytes (CD45.2^+^) gradually increased from 70% at one month to 90% at three months after HSCT (**Figure 2C**). There were no significant differences in the engraftment or the percentages of CD4 T cells, CD8 T cells, and B cells among the 2bMOG_TD_, 2bMOG_1-157_, 2bMOG_FL_, and 2bGFP groups (**Figures 2D–G**). Representative flow cytometry plots are shown in **Supplemental Figure 1**. These results demonstrate that ectopic expression of MOG to platelets does not influence engraftment or hematopoiesis.

**Figure 3:**
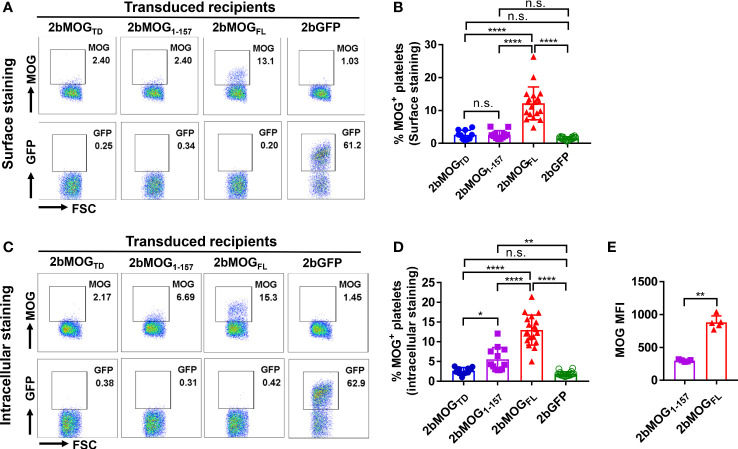
Platelet-MOG expression in 2bMOG-transduced recipients. Blood samples were collected from 2bMOG-transduced recipients after at least 3 weeks of BM reconstitution. Platelets were isolated, stained for CD41 and MOG with or without cell permeabilization, and analyzed by flow cytometry. 2bGFP was used as a control. **(A)** Representative dot plots from flow cytometry analysis by surface staining of MOG expression at 3 weeks after HSCT. **(B)** The percentages of MOG positive platelets in recipients by surface staining are shown. For individual mice analyzed more than once over the study, the average platelet MOG expression was calculated. **(C)** Representative dot plots from flow cytometry analysis by intracellular staining of MOG expression at 3 weeks after HSCT is shown. **(D)** The percentages of MOG positive platelets in indicated recipients by intracellular staining is shown. For individual mice analyzed more than once over the study, the average platelet MOG expression was calculated. **(E)** Representative mean fluorescent intensity (MFI) of intracellular MOG expression in transduced recipients from one trial at 3 weeks after HSCT is shown. MOG MFI was analyzed by flow cytometry analysis through intracellular staining. *P < 0.05; **P < 0.01; ****P < 0.0001. “n.s.” indicates no statistically significant difference between the two groups. **(B, D, E)** Each data point represents one mouse. Data were summarized from four trials. MFI, mean fluorescence intensity.

To investigate 2bMOG protein expression and cellular location, platelets from transduced recipients were stained with anti-MOG antibody with or without permeabilization and analyzed by flow cytometry. As shown in **Figures 3A, B**, MOG was detected on 2bMOG-transduced platelets by surface staining in the 2bMOG_FL_ group with an average level of 12.2 ± 5.0% (n = 19), which was significantly higher than in the 2bMOG_TD_ and 2bMOG_1-157_ groups [2.6 ± 1.4% (n = 10) and 2.6 ± 1.2% (n = 12), respectively]. There were no significant differences in MOG expression on platelets by surface staining between the 2bMOG_TD_ and 2bMOG_1-157_ and the 2bGFP control group (1.5 ± 0.5%, n = 20) (**Figure 3B**).

When platelets were permeabilized, MOG was detected in 13.0 ± 3.8% of platelets in the 2bMOG_FL_ group and 5.6 ± 3.0% in the 2bMOG_1-157_ group, which were significantly higher than in the 2bMOG_TD_ and the 2bGFP groups (2.6 ± 0.9% and 1.8 ± 0.6%, respectively). The MOG positive platelets in the 2bMOG_1-157_ group were significantly lower than in the 2bMOG_FL_ group (**Figure 3D**). There was no significant difference in MOG expression between the 2bMOG_TD_ and 2bGFP groups (**Figures 3C, D**). We further analyzed the mean fluorescence intensity (MFI) of MOG by flow cytometry in 2bMOG_1-157-_ and 2bMOG_FL_-transduced recipients. As shown in **Figure 3E**, the MFI of MOG expression in the 2bMOG_FL_ group was significantly higher than that in the MOG_1-157_ groups (298.4 ± 16.3 *vs*. 881 ± 99.6, respectively, P < 0.01). Together, these data demonstrate that the platelet-specific αIIb promoter-driven MOG fragment expression resulted in differential subcellular localization of MOG protein in platelets. 2bMOG_FL_ lentiviral gene delivery to HSCs resulted in surface expression of MOG by platelets; 2bMOG_1-157_ only introduces intracellular expression, and 2bMOG_TD_ failed to drive MOG expression in platelets.

### Platelet-specific MOG expression induced immune tolerance to MOG

**Figure 4:**
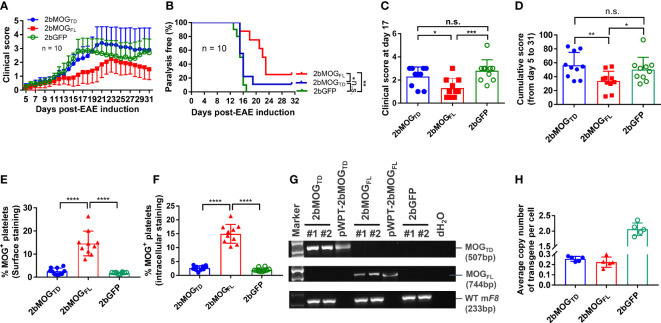
Platelet-specific MOG_FL_, not MOG_TD_, expression ameliorated EAE disease severity. After lentivirus transduction of HSCs followed by HSCT and at least 3 months of BM reconstitution, mice were challenged with MOG_35-55_ peptide emulsified in CFA along with intraperitoneal injection of pertussis toxin on days 0 and 2. Animals were monitored, and clinical scores were recorded during the study period of day 5-31 after EAE induction. **(A)** Clinical scores in 2bMOG_FL_-, 2bMOG_TD_-, and 2bGFP-transduced recipients through the study period after EAE induction. N = 10 in each group. **(B)** The percentage of paralysis-free transduced recipients after EAE induction during the study period of 5-31 days. Mice with a clinical score ≥ 2.5 were defined as having paralysis. N = 10 in each group. **(C)** Clinical scores in transduced recipients at day 17 after EAE induction. **(D)** Cumulative disease scores in transduced recipients during the study period. **(E)** Surface expression of MOG in transduced recipients. **(F)** Intracellular expression of MOG in transduced recipients. **(G)** PCR analysis of MOG proviral DNA in leukocytes from transduced recipients. DNA was purified from peripheral blood leukocytes at 3 weeks after HSCT and the MOG expression cassette was amplified by PCR using primers designed for each construct. WT mouse FVIII was used as an internal control for DNA integrity. **(H)** Quantitative real-time PCR analysis was used to determine the average copy number of LTR in 2bMOG_TD_, 2bMOG_FL_, 2bGFP-transduced recipients. *P < 0.05; **P < 0.01; ***P < 0.001; ****P < 0.0001. “n.s.” indicates no statistically significant difference between the two groups. **(C–F, H)** Each data point represents one mouse. Data were summarized from two trials.

After the BM was fully reconstituted in the recipients, animals were immunized with MOG_35-55_ emulsified in complete Freund’s adjuvant (CFA) combined with pertussis toxin injections to induce EAE. The clinical score from 5 to 31 days after EAE induction was monitored. As shown in **Figure 4A** and **Table 1**, the daily average clinical score in the 2bMOG_FL_ group was significantly lower than in the 2bMOG_TD_ and 2bGFP groups from days 14 to 20. There was no significant difference in the daily average clinical score between the 2bMOG_TD_ and 2bGFP groups. The percentage of paralysis-free animals in the 2bMOG_FL_ group was significantly higher than in the 2bMOG_TD_ and 2bGFP groups, and there was no statistically significant difference between the 2bMOG_TD_ and 2bGFP groups (**Figure 4B**). Seventeen days after EAE induction, the clinical score in the 2bMOG_FL_ group was significantly lower than those in 2bMOG_TD_ and 2bGFP recipients (**Figure 4C**). Similarly, the cumulative disease score in the 2bMOG_FL_ group was significantly lower than in the 2bMOG_TD_ and 2bGFP groups, but there was no difference in the day 17 and cumulative disease scores between the 2bMOG_TD_ and 2bGFP groups (**Figure 4C, D**). These data suggest that platelet-specific MOG_FL_ expression ameliorated EAE disease severity.

**Table 1:** Statistical analysis of the clinical score in 2bMOG_TD_ and 2bMOG_FL_-transduced recipients after EAE induction^†^.

Comparisons of the clinical score in recipients after EAE induction	Days after EAE induction† (Individual P-value)											
	14	15	16	17	18	19	20	21	22	29	30	31
**2bMOG_TD_ *vs*. 2bMOG_FL_**	ns	**	*	*	**	**	**	**	*	*	ns	*
**2bMOG_TD_ *vs*. 2bGFP**	ns	ns	ns	ns	ns	ns	ns	ns	ns	ns	ns	ns
**2bMOG_FL_ *vs*. 2bGFP**	***	***	***	**	**	**	*	ns	ns	ns	*	**

**†**Data not shown for those before 14 days and between days 23-28 as there were no statistically significant differences between the groups. *P < 0.05; **P < 0.01; ***P < 0.001; “ns” indicates no statistically significant difference between the two groups.

There was no detectable MOG protein expression in the platelets from 2bMOG_TD_-transduced recipients, regardless of surface- or intracellular-staining (**Figures 4E, F**), and platelet-targeted MOG_TD_ expression did not affect the development of EAE (**Figures 4A–D**) in mice. To examine cell viability in 2bMOG_TD_-transduced recipients, we used semi-quantitative PCR and quantitative real-time PCR (qPCR) (30) to quantify the MOG proviral DNA. As shown in **Figure 4G**, MOG_TD_ and MOG_FL_ proviral DNA were detected by PCR in the 2bMOG_TD_- and 2bMOG_FL_-transduced recipients, respectively. The copy number of proviral DNA in the 2bMOG_TD_ and 2bMOG_FL_ groups were comparable (**Figure 4H**) when determined by qPCR using primers to amplify the LTR sequence as previously reported (30). These data suggest that the failure of platelet-targeted MOG_TD_ expression in ameliorating EAE is not due to a loss of proviral DNA insertion.

### Eliminating transmembrane domains of MOG expression in platelets enhanced the efficacy in attenuating disease development in EAE

**Figure 5:**
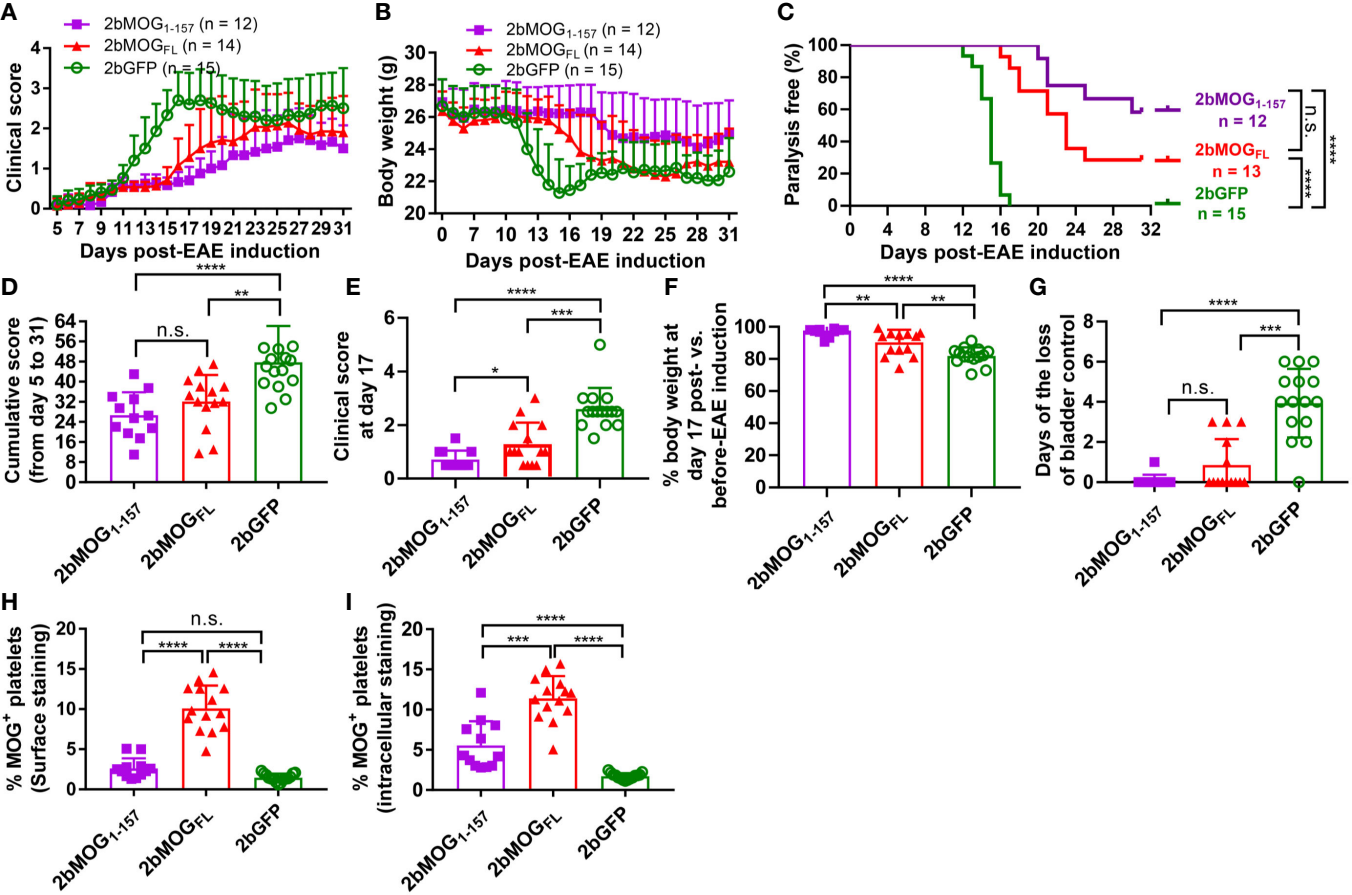
Eliminating MOG transmembrane domains (MOG_1-157_) enhanced clinical efficacy in immune tolerance induction in EAE. After lentivirus transduction of HSCs followed by HSCT and at least 3 months of BM reconstitution, EAE was induced. Clinical scores and body weights were monitored daily during the study period of 5-31 days after EAE induction. Loss of bladder control (urinary incontinence) during days 5-20 was assessed by visual observation of wetness on the animal’s fur on the caudal abdomen. **(A)** The average daily EAE score of 2bMOG_1-157_-, 2bMOG_FL_-, 2bGFP-transduced recipients over time are shown. **(B)** Body weights of 2bMOG_1-157_-, 2bMOG_FL_-, 2bGFP-transduced recipients over time are shown. **(C)** The percentages of transduced recipients that were paralysis free after EAE induction are shown. **(D)** The cumulative scores of 2bMOG_1-157_, 2bMOG_FL_, and 2bGFP recipients up to 31 days after EAE induction are shown. **(E)** The EAE score of 2bMOG_1-157_-, 2bMOG_FL_-, and 2bGFP-transduced recipients at day 17 after EAE induction are shown. **(F)** Body weights of the 2bMOG_1-157_, 2bMOG_FL_, and 2bGFP recipients at day 17 after EAE induction are shown. **(G)** Days of bladder control loss in transduced recipients after EAE induction during the study period are shown. **(H)** Surface expression of MOG in transduced recipients is shown. **(I)** Intracellular expression of MOG in transduced recipients is shown. *P < 0.05; **P < 0.01; ***P < 0.001; and ****P < 0.0001. “n.s.” indicates no statistically significant difference between the two groups. **(D-H, I)** Each data point represents one mouse. Data were summarized from three trials.

We then compared if the cellular location of MOG protein expression in platelets impacted the efficacy of immune tolerance induction in the EAE model. We compared tolerance induction in 2bMOG_1-157_ and 2bMOG_FL_ recipients. As shown in **Figure 5A** and **Table 2**, clinical scores in both the 2bMOG_1-157_ and 2bMOG_FL_ groups were significantly lower than in the 2bGFP control group from day 12 to 19. The clinical score in the 2bMOG_1-157_ group was significantly lower than in the 2bMOG_FL_ group between days 17-19. Between days 20-31 after EAE induction, clinical scores in the 2bMOG_1-157_ group were significantly lower than in the 2bGFP group; however, there were no statistically significant differences between the 2bMOG_FL_ and 2bGFP groups although it appears lower in the 2bMOG_FL_ group. The body weights, another parameter for evaluating EAE disease development, in both the 2bMOG_1-157_ and the 2bMOG_FL_ groups were significantly higher than in the 2bGFP group between days 12-17 (**Figure 5B** and **Table 3**). Between days 18-31, the body weights in the 2bMOG_1-157_ were significant higher than in the 2bGFP group, but there were no differences between the 2bMOG_FL_ and 2bGFP groups. The body weight in the 2bMOG_1-157_ group was significantly higher than the 2bMOG_FL_ group between days 16-26 and 31 (**Figure 5B** and **Table 3**). The clinical scores were negatively correlated with the body weight after EAE induction in all three groups (**Supplemental Figures 2A–C**). During the study period, the number of paralysis-free mice after EAE induction in both the 2bMOG_1-157_ and 2bMOG_FL_ groups was significantly higher than in the 2bGFP control group. It appears that the number of paralysis-free mice after EAE induction in the the 2bMOG_1-157_ group was higher than in the 2bMOG_FL_ group, but there was no statistically significant difference between the two groups (**Figure 5C**).

**Table 2:** Statistical analysis of the clinical score in 2bMOG_1-157_ and 2bMOG_FL_-transduced recipients after EAE induction^‡^ .

Comparisons of the clinical score in recipients after EAE induction	Days after EAE induction‡ (Individual P-value)																
	12	13	14	15	16	17	18	19	20	21	22	23	24	28	29	30	31
**2bMOG_1-157_ *vs*. 2bMOG_FL_**	ns	ns	ns	ns	ns	*	*	*	ns	ns	ns	ns	ns	ns	ns	ns	ns
**2bMOG_1-157_ *vs*. 2bGFP**	***	**	***	****	****	****	****	****	***	*	*	*	*	*	***	**	**
**2bMOG_FL_ *vs*. 2bGFP**	***	***	****	****	***	***	***	***	ns	ns	ns	ns	ns	ns	ns	ns	ns

**‡**Data not shown for those before 12 days and between days 25-27 as there were no statistically significant differences between the groups. *P < 0.05; **P < 0.01; ***P < 0.001; ****P < 0.0001. “ns” indicates no statistically significant difference between the two groups.

**Table 3:** Statistical analysis of the body weight in 2bMOG_1-157_ and 2bMOG_FL_-transduced recipients after EAE induction§.

Comparisons of the body weight in recipients after EAE induction	Days after EAE induction§ (Individual P-value)																			
	12	13	14	15	16	17	18	19	20	21	22	23	24	25	26	27	28	29	30	31
**2bMOG_1-157_ *vs*. 2bMOG_FL_**	ns	ns	ns	ns	*	***	***	**	*	*	**	**	**	***	**	ns	ns	ns	ns	*
**2bMOG_1-157_ *vs*. 2bGFP**	**	***	****	****	****	****	****	****	**	*	**	**	**	**	*	**	*	**	***	**
**2bMOG_FL_ *vs*. 2bGFP**	**	****	****	****	****	**	ns	ns	ns	ns	ns	ns	ns	ns	ns	ns	ns	ns	ns	ns

**§**Data not shown for those before 12 days as there were no statistically significant differences between the groups. *P < 0.05; **P < 0.01; ***P < 0.001; ****P < 0.0001. “ns” indicates no statistically significant difference between the two groups.

The cumulative disease scores from days 5-31 in the 2bMOG_1-157_ and 2bMOG_FL_ groups were 26.7 ± 9.1 (n = 12) and 32.1 ± 10.6 (n = 14), respectively, which were significantly lower than in the 2bGFP group (47.7 ± 14.5) (n = 15) (**Figure 5D**). On day 17, when the disease enters the chronic phase (37), clinical scores in the 2bMOG_1-157_ and 2bMOG_FL_ groups were significantly lower than in the 2bGFP group (**Figure 5E**). Conversely, at day 17, body weights in the 2bMOG_1-157_ group were 97.6 ± 3.3% of the base body weight before EAE induction, which was significantly higher than in the 2bMOG_FL_ group (90.3 ± 7.9%) and the 2bGFP group (81.9 ± 5.4%) (**Figure 5F**). Of note, the cumulative disease score and the score and body weight at day 17 in the 2bMOG_1-157_ group were significantly different from those in the 2bMOG_FL_ group (**Figure 5D-F**). We also monitored animals to assess loss of bladder control between days 5-20 after EAE induction. As shown in **Figure 5G**, only 1 of 12 2bMOG_1-157_-transduced recipients had one day of bladder control loss, and 5 of 14 2bMOG_FL_-transduced recipients had a loss of bladder control varying from 1-3 days. In contrast, 14 of 15 2bGFP-transduced recipients suffered bladder control loss ranging from 2-6 days. The days of bladder control loss in the 2bMOG_1-157_ and 2bMOG_FL_ groups was significantly lower than in the 2bGFP group. In the 2bMOG_FL_ group, 10.1 ± 2.8% of platelets expressed MOG protein surface staining and 11.4 ± 2.8% by intracellular staining, but MOG protein expression could only be detected *via* intracellular staining in the 2bMOG_1-157_ group with a percentage of 5.6 ± 3.0 (**Figures 5H, I**). The platelet-MOG expression negatively associated with clinical score when GFP control mice were included for comparison (**Supplemental Figure 3A**). However, there were no significant correlations between the percentage of MOG-positive platelets and EAE scores within the groups of 2b MOG_1-157_ or MOG_1-157_ transduced mice (**Supplemental Figures 3B, C)**. Collectively, these results demonstrate that both MOG_1-157_ and MOG_FL_ gene transfer to platelets induced immune tolerance to MOG with MOG_1-157_ exhibiting higher efficacy in ameliorating EAE than MOG_FL_ when MOG expression was targeted to platelets.

### MOG_1-157_ and MOG_FL_ induce immune tolerance through different mechanisms

**Figure 6:**
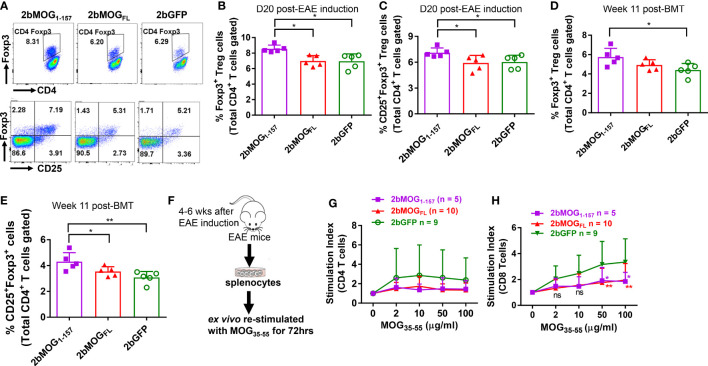
Platelet-targeted MOG_1-157_ expression lead to Treg accumulations and suppressed CD8 T cell recall responses to MOG_35-55_ stimulation. Leukocytes from peripheral blood at 11 weeks after transplantation of transduced HSCs before EAE induction and 20 days after EAE induction were stained for CD4, CD25, and Foxp3 and analyzed by flow cytometry. One to two months after EAE induction, splenocytes from transduced recipients were isolated, labeled with Violet CellTracer, and cultured with various doses (0, 2, 10, 50, and 100 μg/ml) of MOG_35-55_ peptide for 3 days. Cells were harvested and stained for CD4 and CD8. Zombie Red™ staining was used to exclude dead cells. After staining, cells were analyzed by flow cytometry. **(A)** Representative dot plots of flow cytometry analysis of Treg cells in transduced recipients after EAE induction are shown. **(B)** The percentages of Foxp3^+^ Treg cells in transduced recipients after EAE induction are shown. **(C)** The percentages of CD25^+^Foxp3^+^ Treg cells in transduced recipients after EAE induction are shown. **(D)** The percentages of Foxp3^+^ Treg cells in transduced recipients before EAE induction are shown. **(E)** The percentages of CD25^+^Foxp3^+^ Treg cells in transduced recipients before EAE induction are shown. **(F)** The workflow of the T cell proliferation assay is shown. **(G)** The stimulation index of CD4 T cell proliferation in each group cultured with various concentrations of MOG_35-55_ is shown. **(H)** The stimulation index of CD8 T cell proliferation in indicated groups with various concentrations of MOG_35-55_ is shown. The stimulation index (SI) was calculated as follows: SI = (the percentage of proliferating daughter cells in MOG_35-55_-treated wells)/(the percentage of proliferating daughter cells in control wells with 0 μg/ml of MOG_35-55_). Two-way ANOVA was used to compare T cell stimulation indexes among groups. *P < 0.05; **P < 0.01. **(B–E, G, H)** Each data point represents one mouse. Data were summarized from two trials.

Since CD4^+^Foxp3^+^ T regulatory (Treg) cells are important in suppressing EAE development (37–39), we analyzed Treg cells in peripheral blood by flow cytometry. As shown in **Figures 6A–C**, on day 20 after EAE induction, Foxp3^+^ and CD25^+^Foxp3^+^ CD4 Treg in the 2bMOG_1-157_ group were significantly higher than in the 2bMOG_FL_ and 2bGFP groups, but there was no significant difference between the 2bMOG_FL_ and 2bGFP groups. Before EAE induction, Treg cells in the 2bMOG_1-157_ group were significantly higher than in the 2bGFP group, but no statistically significant difference between the 2bMOG_1-157_ and 2bMOG_FL_ groups was observed, although there were trends in both the percentage and total number of Treg cells in the 2bMOG_FL_ group higher than the 2bGFP group (**Figures 6D, E**). These results indicate that platelet-targeted MOG_1-157_ expression can promote Treg cell expansion or induction in 2bMOG_1-157_-transduced recipients and that Treg cells further increased after EAE induction, which likely played an important role in suppressing EAE.

To investigate if T cells from 2bMOG-transduced recipients after EAE induction could still respond to MOG restimulation, we performed T cell proliferation assays (**Figure 6F**). Splenocytes were isolated from animals at least four weeks after EAE induction and *ex vivo* co-cultured with various concentrations of MOG_35-55_ for 72 hours. CD4 and CD8 T cell proliferation was analyzed by flow cytometry. As shown in **Figure 6G**, it appeared that splenic CD4 T cells from the 2bGFP group proliferated more than the 2bMOG_1-157_ and 2bMOG_FL_ groups, but there was no statistically significant difference in splenic CD4 T cell proliferation in response to MOG_35-55_ restimulation among the 2bMOG_1-157_, 2bMOG_FL_, and 2bGFP groups. Interestingly, the proliferation index of CD8 T cells from the 2bGFP group restimulated with MOG_35-55_ peptide in a dose-dependent manner was significantly higher than in the 2bMOG_FL_ and 2bMOG_1-157_ groups (**Figure 6H**). These results suggested that CD8 T cells were primed in EAE induction and MOG specific CD8 T cells can be induced to tolerize to MOG stimulation when MOG expression was targeted to platelets.

## Discussion

Current therapies for patients with MS mainly target the immune system using immune suppressive agents, which have potential side effects due to systematic immune suppression. Developing antigen-specific immune tolerance is an attractive approach for MS treatment. Several strategies, such as peptide-coupled mononuclear cells or splenocytes, antigen-loaded dendritic cells, or engineered Treg cells have been shown to be efficacious in animal models, yet therapeutics for antigen-specific immune tolerance remains an unmet clinical need for patients with MS (40–44). It has been shown that transient depletion of T cells followed by administration of recombinant myelin could silence relapsing EAE when the treatment was initiated in the early stage, but failed to halt progression (45). Here we described a novel approach to induce immune tolerance in EAE *via* lentivirus-mediated platelet-specific gene delivery to HSCs to introduce long-term MOG expression in platelets.

Our previous studies have shown that platelet targeted gene transfer can effectively induce antigen-specific immune tolerance in hemophilia A and B mice even with pre-existing immunity (24, 26–30, 46). The present study provides proof-of concept that platelet-targeted gene therapy approach could be applied to induce immune tolerance in autoimmune disease model EAE. Our findings reveal that long-term immune tolerance could be achieved in an EAE model *via* platelet-targeted MOG expression and that the efficacy of the immune tolerance induction depended on the platelet-MOG expression level and the subcellular location. Full-length MOG protein contains two transmembrane domains and is expressed on the surface of oligodendrocytes (34, 47). The truncated MOG peptide/proteins exhibited different expression levels and subcellular locations depending on the functional domains harbored. We found that when the MOG peptide/protein expression cassette was introduced into the human promegakaryocyte cell line Dami, 2bMOG_FL_ was expressed on the cell surface while 2bMOG_TD_ and 2bMOG_1-157_ were expressed intracellularly. Similar expression patterns were found in platelets from the 2bMOG_1-157_- and 2bMOG_FL_-transduced recipients. However, MOG expression was undetectable in 2bMOG_TD_-transduced platelets, and EAE development was not attenuated in 2bMOG_TD_-transduced recipients. This might be due to the following: (1) MOG_TD_ is a truncated transgene without a signal peptide sequence, which may result in nonsense-mRNA mediated RNA decay *in vivo* or (2) platelets are small (compared to Dami cells), thus, the expression of MOG_TD_ did not reach the threshold of being detected by the assay. The undetectable level of MOG expression in 2bMOG_TD_-transduced platelets might explain the failure to ameliorate EAE in 2bMOG_TD_-transduced recipients.

In 2bMOG_1-157_- and 2bMOG_FL_-transduced recipients, EAE disease development was attenuated compared to 2bGFP-transduced controls, suggesting that platelet-targeted MOG_1-157_ and MOG_FL_ expression can induce immune tolerance to MOG in EAE model mice even though they were primed with MOG together with strong adjuvants (CFA plus pertussis toxin). Although the MOG-positive platelets and MFI of MOG peptides/protein were lower in mice transduced with 2bMOG_1-157_ than 2bMOG_FL_, targeting MOG_1-157_ expression to platelets showed better efficacy in attenuating EAE disease development than 2bMOG_FL_. These data suggest that, to some extent, the efficacy of immune tolerance induction has little to do with the expression level of neoprotein, but that the cellular location in platelets is a critical factor. This conclusion was further evidenced by the findings from our previous platelet-targeted OVA expression study (31). It is known that von Willebrand factor (VWF) propeptide (Vp) can reroute unrelated secretory protein to a storage pathway. In the OVA model study, two lentiviral vectors, which harbored either an OVA expression cassette driven by the αIIb promoter (2bOVA) or Vp-incorporated 2bOVA (2bVpOVA), were used, and the efficacy of immune tolerance induction was compared. While the average platelet-OVA expression levels in 2bVpOVA-transduced recipients were 17-fold lower than in 2bOVA recipients, 2bVpOVA lentiviral gene delivery to HSCs induced OVA-specific immune tolerance was effective as 2bOVA in suppressing anti-OVA antibody production and in preventing skin graft rejection from CAG mice (31). We speculated that, within a certain range of expression levels, the efficacy of immune tolerance induction might not correlate to the expression level of neoprotein, as there are dual mechanisms: peripheral antigen-specific CD4 T cell deletion and Treg cell accumulation (31), that promote immune tolerance in our platelet-targeted gene therapy.

Apart from the expression level of the neoprotein, the subcellular location of the MOG protein was also a major factor in how it impacted immune tolerance induction in platelet-targeted gene therapy. Our previous studies have demonstrated that when FVIII, FIX, or OVA were ectopically targeted to platelets, neoprotein was expressed and stored in platelets, and that platelet-derived neoprotein could effectively induce antigen-specific immune tolerance in treated animals (24, 26, 27, 29, 31). In contrast, when GPIbα or GPIIIa, a platelet membrane protein, was targeted to the surface of the platelet under the same promoter, some of the transduced recipients did produce antibodies against the neoprotein after platelet gene therapy (48, 49). The antibodies in the GPIb model only lasted for three weeks and disappeared subsequently without treatment, indicating immune tolerance could still be established (49). The precise mechanism of why the cellular location of neoprotein impacts immune tolerance induction is still unclear. We speculate that when neoprotein is expressed on the cell surface, it may increase its exposure to the immune system, triggering immune responses if the neoprotein expression level is not sufficient to induce immune tolerance through clonal deletion of antigen-specific CD4 and CD8 T cells. Further studies to understand the underlying mechanisms are warranted. In addition, platelets turnover every 4-5 days in mice, where aged platelets are taken up in the spleen and liver (50). This may lead to more tolerogenic processing of their intracellular contents by splenic/liver macrophages. There is far more inside the platelets than on the membrane, which could be another possibility why neoprotein expressed and stored inside platelets is more tolerogenic than surface expression.

Our previous OVA model studies have demonstrated that long-term antigen-specific immune tolerance is established through dual peripheral tolerance pathways: deletion of peripheral antigen-specific CD4 T cells and induction of antigen-specific Treg cells (31, 32). Using the OVA model, we have shown that the deletion of peripheral antigen-specific CD4 T cells is more prominent in mice with a higher level of platelet-derived OVA expression, whereas with a lower platelet-OVA level but better storage, the increase in antigen-specific Treg cells was dominant (31). In the current study using the MOG model, Treg cells increased in 2bMOG_1-157_-transduced recipients but not in 2bMOG_FL_-transduced recipients. This result partly agrees with the conclusion that expansion of Treg cells dominates in tolerance mechanisms driven by lower levels of platelet-derived neoprotein expression. This could be due to insufficient levels of MOG expressed in 2bMOG_1-157_-transduced recipients to induce antigen-specific CD4/CD8 T cell deletion. However, intracellular platelet-MOG might be effective in inducing antigen-specific Treg cells since platelets contain abundant amounts of TGFβ-1, which induce Foxp3 expression (51). Indeed, our current study shows that the percentage of Treg cells in the 2bMOG_1-157_ group was further increased and significantly higher than in the 2bMOG_FL_ and 2bGFP groups.

Besides antigen-specific immune tolerance induced in CD4 T cells after platelet-targeted gene therapy (27, 31), our recent study using the OVA model revealed that OVA-specific effector CD8 T cells can also be deleted in peripheral lymphoid organs after platelet-specific OVA gene transfer (32). In the present study, *ex vivo* T cell proliferation assays showed that splenic CD8 T cells from the 2bGFP group could proliferate in response to the MOG_35-55_ restimulation, but not the 2bMOG_1-157_ and 2bMOG_FL_ group, suggesting that the MOG-specific CD8 T cells were tolerized in 2bMOG_1-157_- and 2bMOG_FL_-transduced recipients. This could be a potential mechanism of immune tolerance in 2bMOG-transduced recipients during EAE development. While MS has long been considered a CD4 T cell-mediated disease, accumulating evidence has demonstrated that CD8 T cells also play an important role in the human disease of MS and certain mouse models of EAE (52–54). Thus, inducing immune tolerance *via* modulating both CD4 and CD8 T cells may be beneficial in controlling EAE/MS disease development. Our current study demonstrates that platelet-targeted MOG expression induced immune tolerance *via* the Treg and CD8 T cell pathways and that the cellular location of the neoprotein in platelets may govern the tolerization mechanisms. Why the cellular location of neoprotein in our platelet gene therapy results in different clinical efficacy and mechanisms of immune tolerance induction are still unclear. Further studies are needed to illustrate these questions.

In summary, here we evaluated the efficacy of platelet-targeted gene therapy to induce immune tolerance in autoimmune disease model EAE. We found that platelet-targeted MOG_1-157_ and MOG_FL_ expression resulted in different subcellular locations of neoprotein and Treg cells responses. CD8^+^ T cells were tolerized after platelet-MOG gene therapy. Both 2bMOG_1-157_ and 2bMOG_FL_ expressed in platelets ameliorated EAE, but transmembrane-domain-less MOG_1-157_ displayed significantly greater efficacy in inducing immune tolerance and attenuating the development of EAE than full-length MOG_FL_. Our data demonstrate that targeting transmembrane domain-deleted MOG expression to platelets can effectively induce antigen-specific immune tolerance in EAE. Our study suggests that platelet-targeted gene therapy could be a promising approach for the treatment of patients with autoimmune disease MS.

## Materials and methods

### Antibodies and reagents

The following rat anti-mouse monoclonal antibodies (MoAbs) directly conjugated with fluorophore purchased from eBioscience (San Diego, CA, USA) were used in our studies for flow cytometry analysis: CD45.1-FITC, CD45.2-APC eFluor 780, CD4- eFluor 450, CD8-PE Cy7, B220-PerCP Cy5.5, CD25-APC, Foxp3-PE, and the Foxp3 Transcription Factor Staining Buffer Set. Anti-mouse CD42b MoAb conjugated with DyLight-649 was purchased from Emfret Analytics (Eibelstadt, Germany). Rabbit anti-mouse MOG polyclonal antibody was purchased from Aviva System Biology (San Diego, CA, USA). Mouse BD Fc Block was purchased from BD Pharmingen (Franklin Lakes, NJ, USA). BD Cytofix™ fixation buffer was purchased from BD Biosciences (Franklin Lakes, NJ, USA). The EasySep™ Mouse SCA1 Positive Selection Kit was purchased from StemCell Technologies Inc. (Cambridge, MA, USA). The QIAamp DNA Blood Mini Kit was purchased from QIAGEN (Germantown, MD, USA). GoTaq^®^ Green Master Mix was purchased from Promega (Madison, WI, USA). The mouse MOG_35-55_ (MEVGWYRSPFSRVVHLYRNGK) was synthesized by the Protein Chemistry Core laboratory of Versiti Blood Research Institute, Wisconsin, USA. Pertussis toxin was purchased from List Labs (Campbell, CA, USA). CFA was purchased from Chondrex, Inc. (Woodinville, WA, USA).

Tyrode buffer contained 20 mM HEPES, 137 mM NaCl, 13.8 mM NaHCO_3_, 0.36 mM NaH_2_PO_4_, and 2.5 mM KCl. Modified Tyrode buffer was prepared with 5.5 mM glucose, 0.25% BSA, and 1 mM MgCl_2_ in Tyrode Buffer. Platelet collection buffer was prepared with modified Tyrode buffer, 3.8% Sodium citrate, and 50 ng/ml Prostaglandin E1. Gey’s solution was prepared with 155 mM NH_4_Cl and 10 mM KHCO_3_. FACS buffer contained 0.5% BSA and 0.01% NaN_3_ in DPBS.

### Mice

All the animals used in this study were on the C57BL/6 genetic background. Wild-type (WT) CD45.1 and WT CD45.2 mice used in this study were purchased from Jackson Laboratory (Bar Harbor, ME, USA) and maintained in our animal facility. All mice were kept in pathogen-free microisolator cages in the Biomedical Research Center operated by the Medical College of Wisconsin. Isoflurane or Xylazine/Ketamine was used for anesthesia. Animal studies were performed according to a protocol approved by the Institutional Animal Care and Use Committee of the Medical College of Wisconsin.

### MOG vector construction and lentivirus production

Genome information for mouse MOG (NM_010814.2) was obtained from the National Center for Biotechnology Information. The MOG_TD_ (2 copies of MOG amino acids 64-146) and MOG_FL_ (full length of MOG protein) cassettes were synthesized by the GeneArt (Thermo Fisher Scientific, Waltham, MA, USA). The MOG_1-157_ cassette (amino acid of MOG amino acid 1-157) was amplified by PCR using primers, as shown in **Table 4**. The pWPT-2bOVA vector (31), which harbors a platelet-specific promoter αIIb, was used as a backbone vector in this study. The MOG_TD_, MOG_1-157_, and MOG_FL_ cassettes were subcloned downstream of the αIIb promoter in the pWPT-2bOVA vector (31) and replaced the OVA cassette to generate the pWPT-2bMOG_TD_, pWPT-2bMOG_1-157_, and pWPT-2bMOG_FL_ vectors, respectively. The recombinant lentiviruses were produced and titrated following protocols described in our previous report (24, 31).

**Table 4:** The sequences of primers used for PCR to amplify MOG.

Gene	Primers
MOG_TD_	Sense: 5’ ATTCACGCGTGCCATGGAG 3’ Antisense: 5’ CGGGTCGACTCACGCATCTT 3’
MOG_1-157_	Sense: 5’TTCACGCGTGCCATGGCCTGTTTGTG 3’ Antisense: 5’ CGGGTCGACTCAAGTCAGCACACCGGGGTTGACCCAAT 3’
MOG_FL_	Sense: 5’TTCACGCGTGCCATGGCCTGTTTGTG 3’ Antisense: 5’ CGGGTCGACTCATCAAAAGGGG 3’

### HSCs transduction and transplantation

Sca-1^+^ cells were isolated from BM of WT CD45.2 mice using a Mouse SCA1 Positive Selection Kit following the protocol provided by the manufacturer. Sca-1^+^ cells were transduced with lentivirus (pWPT-2bMOG_TD_, pWPT-2bMOG_1-157_, pWPT-2bMOG_FL_ or pWPT-2bGFP lentivirus) following procedures as described in our previous reports (24, 31). After transduction, 1-1.5 x 10^6^ cells per mouse in 250 µL X-VIVO 10 media were transplanted *via* retro-orbital venous injection into 6-week-old WT CD45.1 recipients preconditioned with a 660 cGy total body irradiation using a cesium irradiator (Gammacell 40 Exactor, Best Theratronics, Ltd., Ottawa, Canada). Animals were randomly assigned to the groups 2bMOG_TD_, 2bMOG_1-157_, 2bMOG_FL_ or 2bGFP and transplanted with lentivirus-transduced Sca-1^+^ cells, and referenced as 2bMOG_TD_, 2bMOG_1-157_, 2bMOG_FL_, and 2bGFP recipients, respectively. Blood samples were collected monthly by retro-orbital bleeds with 3.8% sodium citrate anticoagulant (1:10 vol/vol) starting at one month after transplantation, and plasma, leukocytes, and platelets were isolated as previously described (25).

### PCR detection of proviral MOG transgene

For PCR analysis, genomic DNA was purified from peripheral blood leukocytes using QIAamp DNA Blood Mini Kit, and MOG transgene was amplified using GoTaq Green Master Mix. MOG primers were designed to distinguish MOG_TD_ and MOG_FL_ transgenes, and the sequences for primers are listed in **Table 4**. Mouse *F8* (m*F8*) was used as an internal control to confirm DNA integrity. dH_2_O was used a negative control. pWPT-2bMOG_TD_ or pWPT-2bMOG_FL_ plasmid DNA was used as a positive control.

### Flow cytometry analysis

Flow cytometry was used to analyze the chimerism of leukocytes and to determine MOG expression in platelets of transduced recipients. For leukocyte chimerism analysis, leukocytes were isolated from peripheral blood after lysing red blood cells with Gey’s solution. Cells were resuspended in 50 μL of DPBS containing Fc Block and incubated for 10 minutes to block non-specific binding. Cells were then stained with 100 μL of DPBS containing a combination of multiple fluorophore-conjugated antibodies at 4° C for 30 minutes. After staining, cells were washed with 1 mL DPBS, resuspended in 200 μL of FACS buffer, and analyzed by an LSRII flow cytometer (BD Bioscience, Sparks, MD, USA). Samples from WT CD45.1 and WT CD45.2 mice were used as controls. Data were analyzed using FlowJo Software (FlowJo, LLC, Ashland, OR, USA). For intracellular staining for Treg cells, after surface staining, the cells were fixed, permeabilized, and stained using the Foxp3 Transcription Factor Staining Buffer Set, following the protocol provided by the manufacturer.

For flow cytometry analysis of platelet MOG expression, platelets were isolated from peripheral blood and stained for MOG protein expression. Briefly, isolated platelets (1-2 x 10^6^) were fixed with BD Cytofix™ fixation buffer at 4°C for 30 min and permeabilized with 0.5% Triton-X100 for 20 min on ice. The platelets were centrifuged at 1200 g for 5 min, and the platelet pellet was resuspended in 50 μL of 2% normal goat serum in FACS buffer to block non-specific binding for 30 min. Platelets were then stained with primary rabbit anti-mouse MOG polyclonal antibody (5 μg/ml) in FACS buffer for 30 min and a goat anti-rabbit Alexa Fluor^®^ 568 secondary antibody along with an anti-mouse CD42b antibody directly conjugated with DyLight 649 for 30 min at room temperature. After staining, platelets were washed, resuspended in 200 μL of FACS buffer, and analyzed by flow cytometry.

### Mouse EAE induction and disease assessment

After transplantation and full BM reconstitution, which takes ~12 weeks (25), animals were subcutaneously immunized with 200 μg of MOG_35-55_ peptide emulsified in CFA along with the intraperitoneal injection of 200 ng of pertussis toxin on days 0 and 2 following the protocol as described in previous reports (55, 56). Animals were monitored daily from day 5-20 after MOG_35-55_ immunization to assess whether platelet-specific MOG expression could induce immune tolerance and prevent the development of EAE. Mice receiving 2bGFP-transduced HSCs were used as controls in parallel. The signs of EAE were monitored daily using the EAE scoring system (55–57): 0.5 - Partial limp tail; 1 - Limp tail; 1.5 - Complete limp tail with hind limb ataxia; 2 - Limp tail with hind limb paresis; 2.5 - Limp tail with one side of hind limb paralysis; 3 - Limp tail with both sides of hind limb paralysis; 4 - Limp tail, both sides of hind limb paralysis, and forelimb paralysis; and 5 - Moribund/death. Besides monitoring the signs of EAE after MOG_35-55_ immunization, we also monitored body weight. The loss of bladder control (urinary incontinence) was assessed by visual observation of wetness on the animal’s fur on the caudal abdomen.

### *In vitro* T cell proliferation study

One month after EAE induction, splenocytes from transduced recipients were harvested, and red cells were lysed using Red Cell Lysing Buffer. The splenocytes were labeled with CellTrace Violet by CellTrace™ Violet Cell Proliferation Kit. Labeled cells were cultured at 4.5 × 10^5^ cells/well in flat-bottom 96-well plates with 300 μL of completed RPMI 1640 media containing 2, 10, 50, or 100 μg/ml MOG_35-55_. After 72 h culture, the cells were harvested for flow cytometry analysis. Zombie RedTM Fixable Viability Kit staining was used to exclude dead cells. Cells were analyzed by LSRII flow cytometry, and data were analyzed using FlowJo software. T cell proliferation was described by the stimulation index (SI), which was also termed the proliferation index. The stimulation index indicates the fold change in the percentage of proliferating cells after MOG_35-55_ stimulation compared to the condition without MOG_35-55_ stimulation.

### Statistical analysis

All data are presented as the mean ± SD. All EAE clinical score data were evaluated by the nonparametric Mann-Whitney test for two experimental groups or Kruskal-Wallis test for three groups. The incidences of the loss of bladder control between the two experimental groups were analyzed by Fisher Exact test. The log-rank test was used to determine the difference in paralysis free between the groups. The correlation between clinical scores and body weights was determined by the Pearson test. Statistical comparisons of other data sets were evaluated by the unpaired student *t*-test for two experimental groups, and the one-way or two-way ANOVA test for three or more groups. Statistical analysis was performed using GraphPad Prism 8 (GraphPad Software, La Jolla, CA). A value of P < 0.05 was considered statistically significant.

## Data availability statement

The original contributions presented in the study are included in the article/**Supplementary Material**. Further inquiries can be directed to the corresponding author, Dr. Qizhen Shi.

## Ethics statement

This study was reviewed and approved by The Institutional Animal Care and Use Committee of the Medical College of Wisconsin.

## Author contributions

Contribution: YC designed the study, performed experiments, analyzed data, and wrote the manuscript. JS performed experiments and edited the manuscript. WJ and CG performed experiments. CW contributed to the conception of this study. SW was YC’s PhD mentor and provided administrative support for YC. BD helped to design research and edit the manuscript. QS conceived the idea, designed and oversaw research, analyzed data, and wrote the manuscript. All authors contributed to the article and approved the submitted version.

## Funding

This work was supported by a Pilot grant from Children’s Research Institute, Children’s Wisconsin (QS), National Institutes of Health grant HL-102035 (QS), and generous gifts from the Children’s Hospital of Wisconsin Foundation (QS) and Midwest Athletes Against Childhood Cancer and Bleeding Disorders (MACC) Fund (QS).

## Conflict of interest

QS and BD have applied for a US Provisional Patent Application serial no. 650053.00873 entitled ‘‘Immune tolerance induction for autoimmune diseases through platelet targeted gene therapy’’ for the therapy described within this manuscript.

The remaining authors declare that the research was conducted in the absence of any commercial or financial relationships that could be construed as a potential conflict of interest.

## Publisher’s note

All claims expressed in this article are solely those of the authors and do not necessarily represent those of their affiliated organizations, or those of the publisher, the editors and the reviewers. Any product that may be evaluated in this article, or claim that may be made by its manufacturer, is not guaranteed or endorsed by the publisher.
