# Supplementary material for: Targeting transmembrane-domain-less MOG expression to platelets prevents disease development in experimental autoimmune encephalomyelitis
Source: Front Immunol. 2022 Oct 27;13:1029356. doi: 10.3389/fimmu.2022.1029356 (PMC9647046; doi:10.3389/fimmu.2022.1029356)
Supplement: Supplementary file 1 [file DataSheet_1.pdf]

Supplemental Figure 1

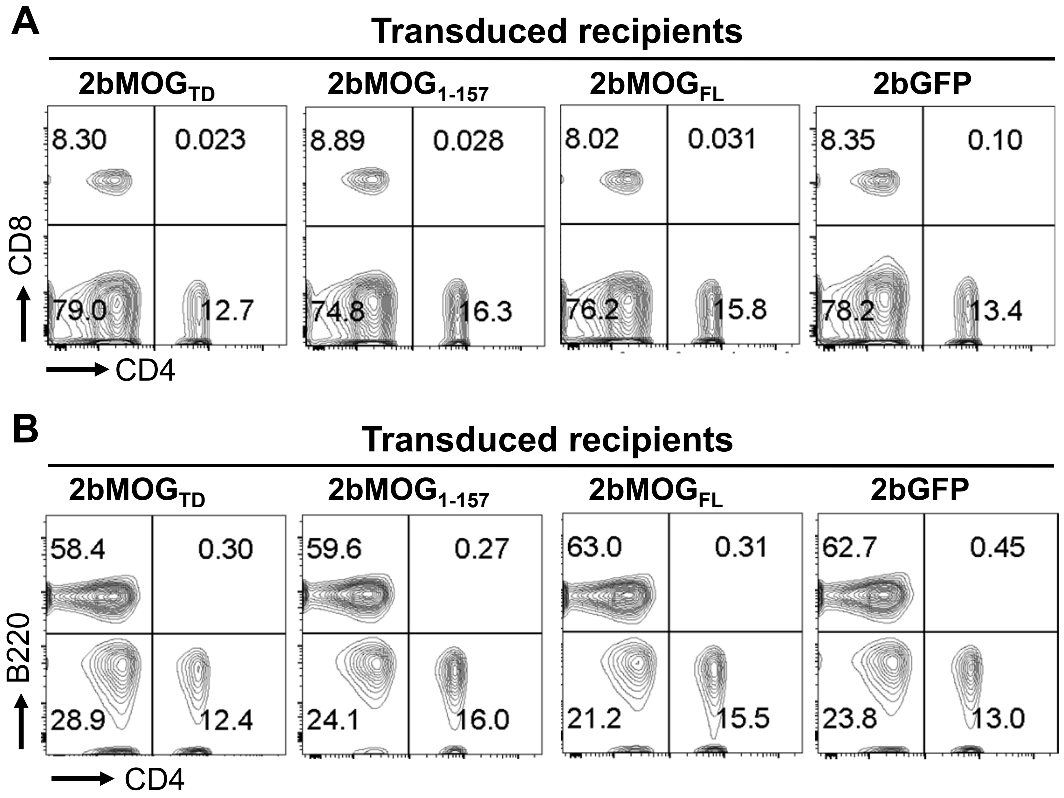

Supplemental Figure 1. Flow cytometry analysis of T and B cells in peripheral blood. Leukocytes were isolated from peripheral blood cells and stained with CD4, CD8, and B220. Representative dot plots from the time point of 7 weeks after BMT are shown. (**A**) The percentages of CD4 and CD8 cells. (**A**)The percentages of B cells.

Supplemental Figure 2

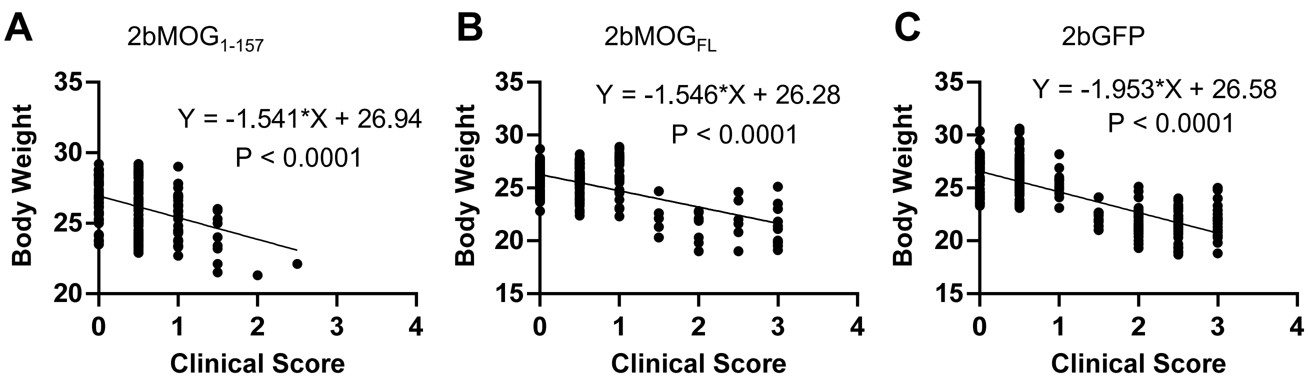

Supplemental Figure 2. The correlation of clinical score and body weight in transduced recipients after EAE induction. After 2bMOG-transduction followed by transplantation and 3 months of bone marrow reconstitution, animals were challenged with MOG_{35-55} peptide emulsified in the complete Freund's adjuvant along with the intraperitoneal injection of pertussis toxin to induce the development of EAE. Animals were monitored daily between day 5-20 after EAE induction for clinical scores and the changes of body weights. The correlation between clinical scores and body weights was determined by the Pearson test. (**A**) The correlation in $2bMOG_{1-157}$-transduced recipients. (**B**) The correlation in $2bMOG_{FL}$-transduced recipients. (**C**) The correlation in 2bGFP-transduced recipients.

Supplemental Figure 3

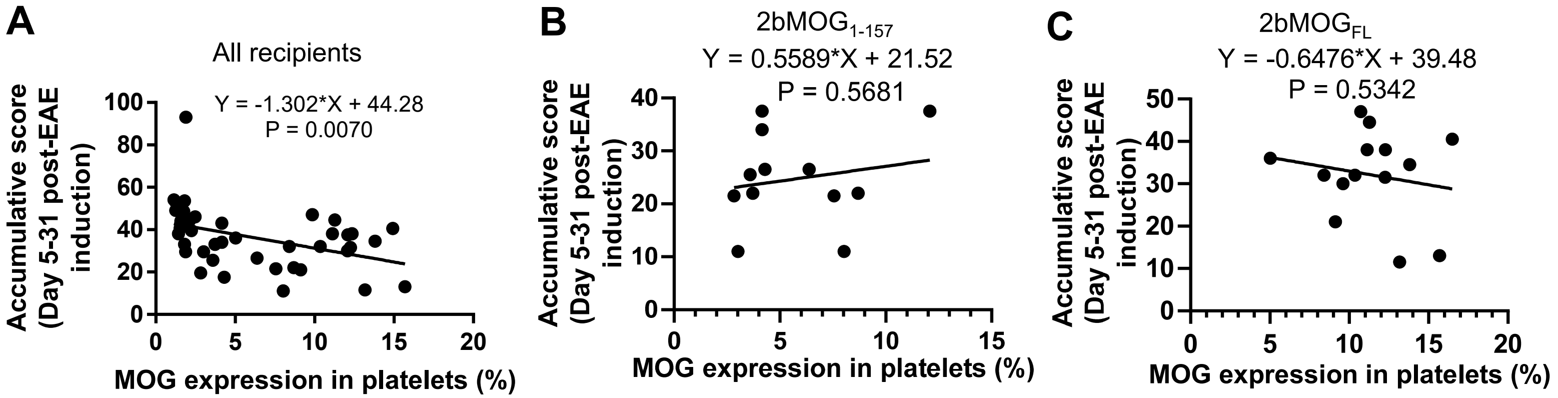

Supplemental Figure 3. The correlation of clinical score and MOG expression in platelets in 2bMOG-transduced recipients after EAE induction. The percentages of MOG positive platelets in transduced recipients were determined by flow cytometry. Data shown were the average platelet-MOG expression from each recipients from at least two time points. After 2bMOG-transduction followed by transplantation and 3 months of bone marrow reconstitution, animals were challenged with $MOG_{35\text{-}55}$ peptide emulsified in the complete Freund's adjuvant along with the intraperitoneal injection of pertussis toxin to induce the development of EAE. Animals were monitored daily between day 5-31 after EAE induction for clinical scores. The correlation between clinical scores and platelet-MOG expression was determined by the Pearson test. (**A**) The correlation in all recipients, including $2bMOG_{1\text{-}157}$-, $2bMOG_{FL}$-, and 2bGFP-transduced recipients. (**B**) The correlation in $2bMOG_{1\text{-}157}$-transduced recipients. (**C**) The correlation in $2bMOG_{FL}$-transduced recipients.
